# No horizontal numerical mapping in a culture with mixed-reading habits

**DOI:** 10.3389/fnhum.2014.00072

**Published:** 2014-02-24

**Authors:** Neda Rashidi-Ranjbar, Mahdi Goudarzvand, Sorour Jahangiri, Peter Brugger, Tobias Loetscher

**Affiliations:** ^1^Faculty of Medicine, Alborz University of Medical SciencesKaraj, Iran; ^2^Department of Cognitive Science, University of TrentoTrento, Italy; ^3^Department of Neurology, University Hospital ZurichZurich, Switzerland; ^4^Zurich Center for Integrative Human Physiology (ZIHP), University of ZurichZurich, Switzerland; ^5^School of Psychology, Flinders UniversityAdelaide, SA, Australia

**Keywords:** cross-cultural, random number generation, mental number line, embodied numerical cognition, automatic processing, line bisection, visuo-motor behavior

## Abstract

Reading habits are thought to play an important role in the emergence of cultural differences in visuo-spatial and numerical tasks. Left-to-right readers show a slight visuo-spatial bias to the left side of space, and automatically associate small numbers to the left and larger numbers to the right side of space, respectively. A paradigm that demonstrated an automatic spatial-numerical association involved the generation of random numbers while participants performed lateral head turns. That is, Westerners have been shown to produce more small numbers when the head was turned to the left compared to the right side. We here employed the head turning/random number generation (RNG) paradigm and a line bisection (LB) task with a group of 34 Iranians in their home country. In the participants’ native language (Farsi) text is read from right-to-left, but numbers are read from left-to-right. If the reading direction for text determines the layout of spatial-numerical mappings we expected to find more small numbers after right than left head turns. Yet, the generation of small or large numbers was not modulated by lateral head turns and the Iranians showed therefore no association of numbers with space. There was, however, a significant rightward shift in the LB task. Thus, while the current results are congruent with the idea that text reading habits play an important role in the cultural differences observed in visuo-spatial tasks, our data also imply that these habits on their own are not strong enough to induce significant horizontal spatial-numerical associations. In agreement with previous suggestions, we assume that for the emergence of horizontal numerical mappings a congruency between reading habits for words and numbers is required.

## Introduction

Our thoughts, perception and actions are shaped by the culture in which we live. Our way of thinking, for example, depends on the social systems we grew up with. That is, East Asians tend to reason in a holistic way, while Westerners exhibit a more analytical thinking style (Nisbett et al., 2001). Different cultural habits also modulate how we perceive things. Italians, for example, judge soccer goals more beautiful when presented with a left-to-right compared to right-to-left trajectory, whereas Arabic speakers show the opposite directional bias (Maass et al., 2007). Our cultural background might also determine motor actions, such as whether we preferably turn our head to the left or right side for kissing somebody on the lips (Shaki, 2013).

Differences in reading directions are thought to play an important role in the emergence of cultural differences in visuo-spatial tasks (Kazandjian and Chokron, 2008). When bisecting horizontal lines, for example, left-to-right readers bisect slightly to the left of the line’s true center. Right-to-left readers, on the other hand, have been reported to misplace the bisection mark to the right of the line’s center (Chokron and Imbert, 1993; Chokron et al., 1997). Similarly, reading direction predicts whether one attends to rightward or leftward features of chimeric faces (Vaid and Singh, 1989), while writing direction can determine whether the trajectory of an apparent motion is perceived as moving to the left or right side (Tse and Cavanagh, 2000).

Reading direction might not only modulate performance in visuo-spatial tasks, but may also influence the way numbers are represented and processed. Preliminary evidence for such influences was reported by Dehaene et al. (1993). In a series of experiments the authors first established that French readers spontaneously mapped small numbers to left and larger numbers to right-sided response codes (the SNARC effect). In their Experiment 7, the authors then showed that a group of Iranians, who had immigrated to France, showed a weaker SNARC effect than French participants. Intriguingly, the time since immigration was related to the direction of the SNARC effect. Iranians with a longer exposure to left-to-right reading direction tended to show a regular SNARC effect, while those Iranians with less familiarity with this reading direction tended to show a reversed SNARC effect -with larger numbers being associated with the left hand. The finding in this study implied a congruency between reading direction for *words* and the representational layout of small to large numbers. It is important to note here that the Iranians’ native language, Farsi, is a mixed-reading language. That is, words in Farsi are written/read from right-to-left, but numerals from left-to-right. Therefore the above experiment suggests that the reading direction for words, and not the one for numerals, determines the mapping between numbers and space.

Subsequent studies provided further evidence for a link between the direction of number representations and reading habits (see Göbel et al., 2011 for a review). Zebian (2005) showed, for example, that Arabic monolingual right-to-left readers associate large and small numbers with the left and right sides of space, respectively. This reversed SNARC effect was significantly reduced in bilingual Arabic participants fluent in right-to-left and left-to-right reading languages. It has been suggested that being fluent in languages with opposite reading habits could weaken spatial-numerical associations (Göbel et al., 2011). Importantly, links between reading direction and spatial-numerical mappings are not restricted to SNARC paradigms, but are also found with other paradigms tapping into spatial-numerical representations, such as bisection tasks (e.g., Kazandjian et al., 2010).

Research on the effects of reading habits also provides ample evidence that the direction of spatial-numerical mapping is flexible and hinges on recently processed stimuli. Bilingual Russian-Hebrew readers, for example, showed a regular SNARC effect after reading a left-to-right Cyrillic script, but they exhibited a significantly reduced effect after reading a right-to-left Hebrew script (Shaki and Fischer, 2008; see also Fischer et al., 2010). In the same vein, Hung et al. (2008) demonstrated that the orientation of the mental number line depends on the task’s context. Chinese readers mapped Arabic numerals on a left-to-right oriented number line, but associated Chinese number words with a vertical, top-to-bottom oriented number line. That is, depending on the format of the numerical notation the spatial-numerical associations differed (Hung et al., 2008).

A wide range of different paradigms have been used to investigate spatial-numerical interactions in Western cultures (see Dehaene and Brannon, 2011). One of those paradigms simply requires participants to generate sequences of random numbers (Loetscher and Brugger, 2007). Studies using random number generation (RNG) paradigms have demonstrated that Westerners implicitly associate the generation of small and large numbers with the left and right side of space, respectively (Hartmann et al., 2012; Vicario, 2012; Di Bono and Zorzi, 2013; Grade et al., 2013). It has been shown, for example, that participants tend to shift their gaze slightly leftward when randomly naming a small number. Rightward gaze shifts, on the other hand, are accompanied with the generation of larger numbers (Loetscher et al., 2010). An analogous pattern of results is found when participants generate random numbers while performing lateral head turns. That is, more small numbers are produced when the head is turned to the left compared to right side turns (Loetscher et al., 2008).

In light of the above findings it is surprising that RNG tasks have never been used to asses spatial mappings of numbers in cultures with right-to-left reading habits. The goal of the current research was to fill this gap. We set out to investigate the spatial representations of numbers in Iranians with an RNG paradigm. For this purpose we replicated the head turning paradigm used by Loetscher et al. (2008). As in the original study, participants were required to rhythmically turn the head from one side to the other while generating random numbers. If the reading direction for words determines the layout of spatial-numerical mappings we expected to find more small numbers after right than left head turns. Such a finding would imply that Iranians code smaller numbers to the right and larger number to the left side of space—the opposite pattern reported by Loetscher et al. (2008) in Western participants. An alternative prediction is that Iranians will show no effect of lateral head turns on the magnitude of generated numbers. Writing/reading directions differ in their native language, Farsi, for words (right-to-left) and numerals (left-to-right). These two opposing habits may cancel one another out. Support for the prediction of a null-finding derives from a study conducted with Israeli participants. Hebrew is also a mixed-reading language, with opposite reading directions for words and numbers, and the participants did not exhibit a reliable spatial association for numbers in a SNARC paradigm (Shaki et al., 2009). Finally, there is also the possibility that small/left and large/right associations as for Westerners are found. This would be an indication that the reading direction for numerals, and not words, is dominant in determining the orientation of the mental number line.

In addition to assessing spatial-numerical association, we also measured visuo-spatial biases in a manual line bisection task (LB task). Based on the previous literature we here expected to find a slight bias to the right of the line’s true center (Chokron and Imbert, 1993; Chokron et al., 1997). Comparing the performances in the visuo-spatial and numerical tasks allowed us to comment on the effect of reading habits in these tasks.

## Materials and methods

### Participants

Thirty-four Iranian men with a mean age of 24 (SD = 7.5) participated in this study. The 25 right and 9 left-handed participants (Chapman and Chapman, 1987) were mostly students and without history of neuropsychiatric or neurological disorder (Campbell, 2000). The higher representation of left-handed participants (26%) in our sample than the proportion of left-handers found in the general population (around 10%, Nicholls et al., 2013) was due to a selection bias. Initially, it was planned to recruit an equal number of right-handers and left-handers for the current experiment. However, this proved to be unachievable due to the difficulty of recruiting left-handed participants. Nevertheless, given the relatively high number of left-handed participants we incorporated handedness as a factor in the analyses.

The native language for all participants was Farsi. All participants had regular English classes in school. They all described themselves as “beginners” and not fluent in any language with a left-to-right reading direction.

The study was approved by the Medical Sciences Ethics Committee of the Alborz University.

### Tasks

The RNG task was as described in Loetscher et al. (2008). Participants were asked to name numbers between 1 and 30 in a sequence as random as possible. With their eyes closed, participants generated a new number every 2 s. The speed of generation was controlled with a metronome running at 0.5 Hz. As in the original study there were two counterbalanced conditions. In the baseline condition, 40 responses were generated while the head was kept straight ahead. In the head-turning condition, participants performed rhythmic head turns to the left and right side, respectively. After participants turned their head about 80^°^ to one side they named a number and then started to turn the head to the opposite side again. The rhythmic head turns continued until a total of 80 numbers, 40 for either direction, were recorded by the examiner. Numbers between 1 and 15 represent “small” numbers in the number space ranging from 1 to 30, those from 16 to 30 represent “large” numbers. The dependent variable was the number of “small” numbers generated.

In the LB task, participants were asked to bisect nine horizontal lines using a pen with their dominant hand. Each line was presented on a separate A4 sheet and measured 160 mm. The dependent variable was the average deviation from the lines’ true center in mm—with positive values indicating a rightward deviation and negative values a leftward deviation.

## Results

The number of “small” numbers was submitted to a repeated-measure ANOVA with *Condition* (left turn, baseline, right turn) as a within-subjects factor and *Handedness* (left, right) as a between-subjects factor. The analysis revealed neither a main effect for *Condition* (*F*_(2,64)_ = 0.17, *p* = 0.85) nor for *Handedness* (*F*_(1,32)_ = 0.32, *p* = 0.57). The interaction between *Condition* and *Handedness* was not significant either (*F*_(2,64)_ = 0.73, *p* = 0.49). Due to its theoretical importance for the current study we also directly compared the number of generated “small” numbers during left and right head turns. Paired *t*-tests revealed no significant differences in the number of “small” numbers between lateral head turns (all participants: *t*_(33)_ = 0.65, *p* = 0.52; only right-handers: *t*_(24)_ = 0.91, *p* = 0.37; only left-handers: *t*_(8)_ = −0.21, *p* = 0.84).

**Figure 1:**
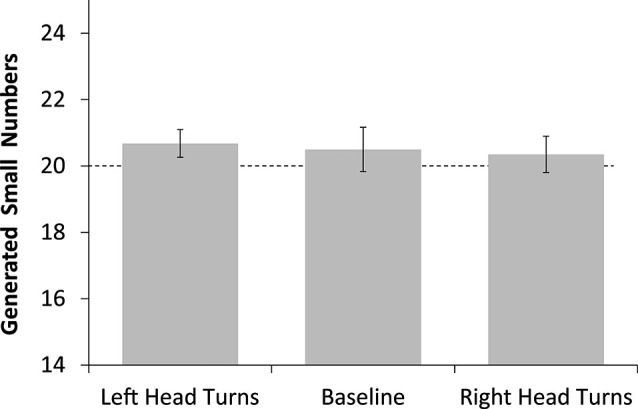
**Number of generated “small” numbers after head turns to the left, a baseline condition (head facing straight ahead), and after head turns to the right**. The dashed line indicates chance expectation.

One sample *t*-tests were conducted to investigate whether there was a bias for naming too many “small” numbers in any of the three conditions. As there was no handedness effect in the ANOVA, data were collapsed across this factor for this analysis. The number of “small” numbers generated did not differ from the expected value of 20.0 in any of the three conditions (*t*_(33)_ < 1.61, *p* > 0.11, see Figure 1). Also, the average of small numbers generated across the three conditions was not significantly different from 20.0 (*t*_(33)_ = 1.21, *p* = 0.23).

The subjective midpoint in the LB task was shifted 0.93 mm (SEM = 0.35) to the right side of the lines’ true center. A one sample *t*-test comparing the participants’ mean deviation to 0 indicated that there was a significant rightward bias (*t*_(33)_ = 2.67, *p* < 0.02) for the 34 participants. The performance in the LB task differed between left (mean deviation: −0.22 mm, SEM = 0.13) and right-handers (mean deviation: 1.34 mm, SEM = 0.44; *t*_(32)_ = 2.08, *p* < 0.05). One sample *t*-tests showed that right-handers deviated significantly to the right of the true center (*t*_(24)_ = 3.02, *p* < 0.01) and that there was no LB bias for left-handers (*t*_(8)_ = −1.79, *p* > 0.11).

The bias in the LB task was not related to the average magnitude of generated “small” numbers across all three conditions (*r* = 0.21, *p* = 0.23).

## Discussion

The study aimed to investigate the spatial mappings of numbers in a culture in which words are read and written from right to left, but numerals from left to right (“mixed-reading habit”). A paradigm that revealed an automatic mapping of small and large numbers to left and right head turns respectively in Westerners (Loetscher et al., 2008) was applied to 34 Iranian participants. In contrast to Westerners, the generation of small or large numbers by Iranians was not modulated by lateral head turns. That is, there was no association of numbers with space, and hence, no evidence for an embodied representation of numbers (Fischer and Brugger, 2011).

The lack of an automatic mapping of numbers in space is in agreement with the few studies that investigated spatial-numerical associations in Iranians. Dehaene et al. (1993), for example, found a weakened SNARC effect in Iranians who had immigrated to France. Our study corroborates these findings by showing that no associations are found when data is collected in Iran, with participants who have been less exposed to Western culture than those in the studies which rely on immigrated participants. Nonetheless, all our participants had some interaction with Western culture. While these interactions were probably less extensive than in previous studies (e.g., Dehaene et al., 1993), we cannot rule out the possibility that they were sufficient to affect the association between numbers and space in the current task. The current study design does not allow disentangling the effects of Western culture exposure and native reading habits on the results. It is noteworthy, however, that even when exposed to Western cultures on a daily basis, native right-to-left readers continue to show specific spatial biases in mental representations (Maass and Russo, 2003).

The reading directions of words (right-to-left) and numbers (left-to-right) differ in Farsi. Our working hypothesis is that these opposite reading habits lead to the disappearance of any preferred lateral association of numbers along the horizontal mental number line. Hebrew readers also use opposite reading directions for words and numbers, and these readers also lacked reliable spatial-numerical associations in a SNARC paradigm (Shaki et al., 2009). It seems reasonable to propose therefore that horizontal associations between numbers and space might only become significant if the reading directions of words and numbers are consistent (Shaki et al., 2009).

Although cultures with mixed-reading directions do not evidence a significant horizontal representation of numbers, it is important to point out that this does not imply the lack of any spatial-numerical mappings in these cultures. The current null-finding, for example, might be the consequence of two conflicting horizontal mappings that cancel each other out. While this idea needs to be further investigated, it has previously been shown that participants with mixed-reading directions (monolingual Israelis) exhibit a radial spatial-numerical mapping when response buttons in a SNARC paradigm were placed in a radial instead of the conventional horizontal arrangement (Shaki and Fischer, 2012). This first demonstration of a spatial-numerical mapping in a mixed-reading culture corroborates the idea that these mappings are flexible and can vary within the same participant depending on the situational context and task demands (Bachtold et al., 1998; Hung et al., 2008; van Dijck et al., 2009; Fischer et al., 2010; Shaki and Fischer, 2012). It seems noteworthy that task demands not only affect spatial-numerical mappings, but also mappings in other dimensions such as space and words (Thornton et al., 2013), or numbers and time (Nicholls et al., 2011). The observation of mappings between word meaning (“moon”) and space (“upper visual space”), for example, is contingent on task demands as it depends on the arrangement of response buttons (Thornton et al., 2013).

Participants’ handedness only affected performance in the visuo-motor LB task, but not in the RNG task. This finding is consistent with previous research. Differences between left and right-handers in LB tasks are commonly observed (Sampaio and Chokron, 1992; Jewell and McCourt, 2000), while handedness seems to be unrelated to spatial-numerical associations (Dehaene et al., 1993; Fischer, 2008).

The observed rightward shift in the LB task is analogous to that described in previous studies assessing visuo-spatial biases in right-to-left reading cultures (Chokron and Imbert, 1993; Chokron et al., 1997), but opposite to the leftward shift found in left-to-right reading cultures (Jewell and McCourt, 2000). Thus, the current results are consistent with the suggestion that reading habits for text play an important role in the cultural differences observed in visuo-spatial tasks (Kazandjian and Chokron, 2008; Kazandjian et al., 2010). However, our data also suggest that these habits on their own are not strong enough to induce significant horizontal spatial-numerical associations. In accord with the conclusions of Shaki et al. (2009) we assume that for the emergence of horizontal spatial mappings a congruency between reading habits for text and numbers is required.

## Conflict of interest statement

The authors declare that the research was conducted in the absence of any commercial or financial relationships that could be construed as a potential conflict of interest.
